# Fabrication of complete titania nanoporous structures via electrochemical anodization of Ti

**DOI:** 10.1186/1556-276X-6-332

**Published:** 2011-04-13

**Authors:** Ghafar Ali, Chong Chen, Seung Hwa Yoo, Jong Min Kum, Sung Oh Cho

**Affiliations:** 1 Department of Nuclear and Quantum Engineering, Korea Advanced Institute of Science and Technology (KAIST), 373-1 Guseong, Yuseong, Daejeon 305-701, Republic of Korea

## Abstract

We present a novel method to fabricate complete and highly oriented anodic titanium oxide (ATO) nano-porous structures with uniform and parallel nanochannels. ATO nano-porous structures are fabricated by anodizing a Ti-foil in two different organic viscous electrolytes at room temperature using a two-step anodizing method. TiO_2 _nanotubes covered with a few nanometer thin nano-porous layer is produced when the first and the second anodization are carried out in the same electrolyte. However, a complete titania nano-porous (TNP) structures are obtained when the second anodization is conducted in a viscous electrolyte when compared to the first one. TNP structure was attributed to the suppression of F-rich layer dissolution between the cell boundaries in the viscous electrolyte. The structural morphologies were examined by field emission scanning electron microscope. The average pore diameter is approximately 70 nm, while the average inter-pore distance is approximately 130 nm. These TNP structures are useful to fabricate other nanostructure materials and nanodevices.

## Introduction

Macro-, nano-, and meso-porous structure gained a lot of attention of the scientific community in the last few decades due to their unique properties and potential application in various fields [1-4]. Particular attention was paid to the self-organized porous materials due to their self-ordered structure and ease of fabrication. One of the most extensively investigated porous materials is porous anodic alumina (PAA) [5]. Highly ordered nano-porous structure can be fabricated on pure aluminum under optimized conditions via two-step electrochemical anodization [6]. PAA are being used mostly as a membrane [7], as a biosensor [8], and as a template for fabrication of secondary nano-meter scale materials [9]. Nano-porous structure formation on other value metals like Zr, Nb, Ta, W, Fe [10], and Al-Ti [11] alloy have been reported by Patrick and co-workers. The next porous material after aluminum which attracted the interest of researchers around the world in the last decade is titanium di-oxide due to the pioneer work of Fujishima and Honda [12] and Regan and Graztal [13].

Titanium di-oxide (TiO_2_, titania) is a semiconductor material and find their application in many areas like self-cleaning [12], solar cell [13,14], photocatalysis [15], drug delivery [16], biomedical implant [17], and sensing [18]. TiO_2 _nano-porous structure (TNP) was first reported by Zwelling et al. [19] via anodization of Ti and Ti alloy in chromic-HF electrolyte. Soon after, Grimes et al. [20] also reported TiO_2 _nanoporous structure in HF-containing aqueous electrolyte with limited thickness. Since then TiO_2 _nanostructure is the main focus of research. Among the various methods of TiO_2 _nanostructure fabrication, anodization is usually known a simple, versatile, and economical one. The nanotubes diameter, length, and smoothness can be easily controlled by varying the electrochemical parameters [21]. TiO_2 _nanotubes have been fabricated in different electrolytes via anodization of pure Ti [22]. A great breakthrough in the fabrication of TiO_2 _nanotubular structure was achieved by Macak et al. [23], and Grimes and co-workers [24], where they reported very smooth, regular, and very long nanotubes in organic viscous electrolytes. A lot of papers have been published so far on the morphologies and applications of TiO_2 _nanotubes. However, very little attention was paid to TiO_2 _nanoporous structure. TNP film was reported by Bu et al. [25] on glass substrate in polyethylene glycol (PEG) using sol-gel method; however, the pore diameter and pore density was not uniform. Beranek et al. [26] and Macak et al. [27] also fabricated TNP in H_2_SO_4_-HF and Na_2_SO_4_-NF electrolytes, respectively, through anodization of Ti. However, from their SEM results, the morphologies of TiO_2 _nanostructure are similar to tubular structure instead of porous structure. Choi et al. [28] also reported TNP structure at the top surface of Ti via nano-imprint and successive anodization of Ti. TiO_2 _nanoporous structures on Si substrates have also been reported by Yu et al. [29] via anodization, but they did not obtain well-defined and ordered pore morphologies. Zhang et al. [30] applied multi-step (3-step) anodization approach to Ti and obtained highly ordered TNP structure only at the top surface after third anodization. According to their report, ordered nano-porous titania showed much higher photocurrent when compared to titania nanotubes due to efficient separation of photo-generated electron-hole pair by nano-porous titania. Very recently, Patrik and co-workers [31] obtained TNP structure under optimized conditions. Although they successfully obtained TNP structure not only on the top surface, but also cross-sectional wise; however, the degree of ordering and uniformity of channels was not achieved. Hence an ideal nanoporous structures like PAA is scarcely obtained.

Here, in this study, we obtained highly orientated TNP structures with uniform and parallel nano-channels using a two-step anodizing method. By changing the nature of electrolyte during second-step anodization, we obtained different morphologies of TiO_2 _nanostructures. Furthermore, we also studied the effect of various electrolytes and prolonged anodizing time on the pore morphology during second-step anodization.

### Experimental procedure

Titanium foil (Ti, Goodfellow, 0.1 mm thickness, 99.6% purity), ammonium fluoride (NH_4_F, Sigma-Aldrich, Germany, 98+%), hydro-fluoric acid (HF, Sigma-Aldrich, Germany , 98+%), ethylene glycol (Extra pure, Junsei Chemical Co. Ltd. Japan), and glycerol (Extra pure, Junsei Chemical Co. Ltd. Japan) are used in their as-received form without further treatment.

Highly ordered and smooth TiO_2 _nanotubes were fabricated by anodization of Ti foils in ethylene glycol (EG) electrolyte containing 0.5 wt% NH_4_F and 0.2 wt% H_2_O. Briefly before anodization, the Ti foils were degreased by sonicating in acetone, isopropyl alcohol, and methanol each for 10 min. Subsequently, the Ti foils were rinsed many times with deionized (DI) water and dried in gas stream. Two electrodes system with Ti-foil as a working electrode and a platinum gauze (15 × 25 × 0.2 mm^3^) as a counter electrode was used for anodization. The first-step anodization was carried out at 50 V in the above-mentioned electrolyte for 7 h using DC power supply system, producing highly ordered and smooth TiO_2 _nanotubes. It is worth mentioning that in this study the first nanotubes layer was separated from the underlying Ti substrates with the help of N_2_-blowing technique instead of using an ultrasonic treatment [32]. This method not only provides a very clean, smooth, uniform, and oriented honeycomb-like a patterned substrates for further anodization but also helps to avoid possible mechanical damage to the substrates. Thus, as a result a high-quality TiO_2 _nanotubes arrays have been achieved. In order to study the effect of electrolytes on pore morphology, a set of experiments were performed in different electrolytes during the second-step anodization. The second-step anodization was conducted in the same EG-based and 0.5 wt% HF aqueous electrolytes under identical parameters for 20 h, producing TiO_2 _nanotubes covered with a thin nanoporous layer on the top surface. The second-step anodization conducted in an electrolyte consisting of glycerol with 0.5 wt% NH_4_F and 0.2 wt% H_2_O under identical parameters for 20 h led to a highly oriented TNP structure.

In addition, we also investigated the effect of anodizing time on the surface topologies of TiO_2 _nanotubes (TNT) and TNP structures. On the basis of our field emission scanning electron microscope (FESEM, Hitachi S-4800, Tokyo, Japan) results, EG and glycerol-based electrolytes were employed for further experiments. Two samples were anodized in the same EG-based electrolyte for different times (11 and 72 h) under identical parameters with the first-step anodization. In another set of experiment, one sample was first anodized in the same EG-based electrolyte and then re-anodized in the same glycerol-based electrolyte for 72 h via the second-step anodization. The structural morphology of the samples was characterized with the help of FESEM attached with energy dispersive X-ray spectroscopy (EDX). The cross-sectional studies were carried out on mechanically cracked samples.

## Results and discussion

**Figure 1:**
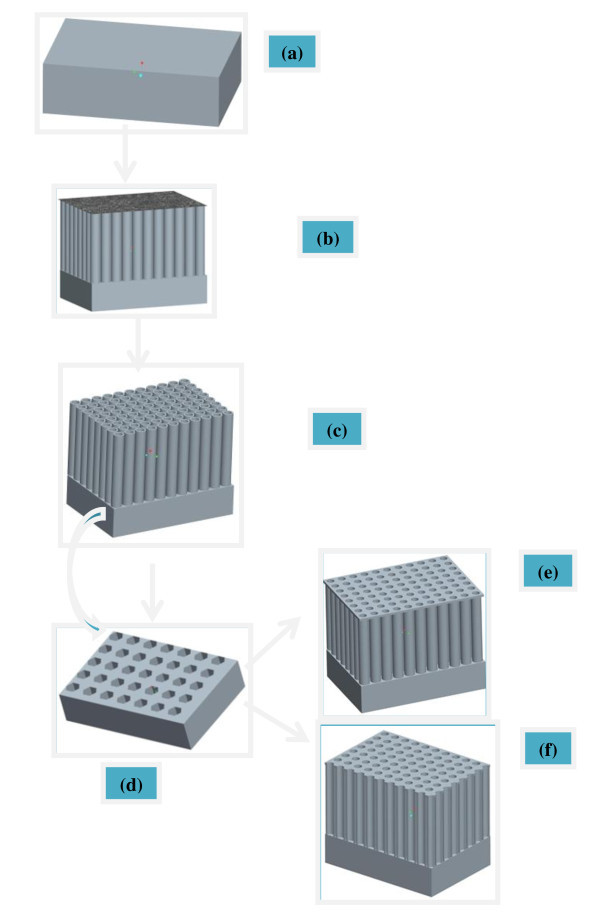
**Schematic of fabrication process of obtaining TiO_2 _nanotubes with nanoporous layer on top and complete titania nanoporous (TNP) structure via two-step anodization**: (a) Ti-foil, (b) first anodization and formation of TNTs with oxide layer on top, (c) TNTs with clear top end, (d) Ti-substrate after separation of TNTs, (e) TNTs covered with thin nanoporous layer, (f) complete TNP structure with uniform and parallel nanochannels.

The formation mechanism of TiO_2 _nanotubular and TNP structure is shown in Figure 1. A well-known two-step anodization method was applied to obtain highly ordered TNT and TNP structure. TNT is fabricated in EG-based electrolyte through the first-step anodization using Ti-foil (Figure 1a). The top surface of the TNT is always covered with some kind of oxide layer (Figure 1b) irrespective of the anodizing time. The oxide layer can be removed with ultrasonic agitation and TNT with clear top end can be achieved (Figure 1c). TNT can be easily peeled-off from under lying Ti-sheet by applying N_2 _stream. Honeycomb-like patterned Ti-substrate is available for further anodization after the separation of TNT from underlying Ti-foil (Figure 1d). The second-step anodization in EG-based and HF-containing aqueous electrolytes produced TNT covered with a thin nano-porous layer on the top surface (Figure 1e), while the second-step anodization in glycerol-based electrolyte led to highly uniform and ordered TNP morphology (Figure 1f).

### The first-step anodization in EG-based electrolyte

**Figure 2:**
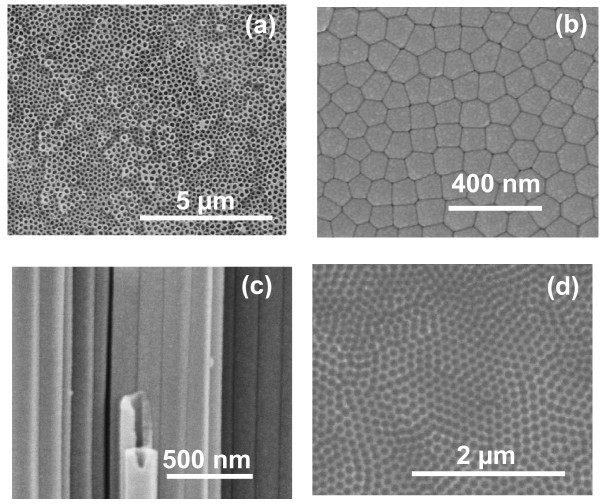
**FESEM images of TiO_2 _nanotubes fabricated in EG containing 0.5 wt% NH_4_F and 0.2 wt% H_2_O via first-step anodization**: **(a) **top surface view, **(b) **bottom surface view, **(c) **cross-sectional view, and **(d) **top view of Ti-substrate after separation of TiO_2 _nanotubes.

Figure 2 shows FESEM images of TNT fabricated in EG-based electrolyte at 50 V for 7 h after first-step anodization. TNT with open mouth-tube morphology was obtained after optimized ultrasonic agitation (Figure 2a). Figure 2b shows the bottom surface morphology of TNT after peeling-off from underlying Ti-substrate. It is clear from the image that TNTs are closed at bottom surface. Figure 2c shows the cross-sectional image of TNT. The image clearly reveals that TNT are very smooth (ripples free) and well-ordered with closed packed morphology, which is consistent well with the bottom surface of TNT (Figure 2b). Ti-substrate after removal of TiO_2 _nanotubes, formed in the first-step anodization, is shown in Figure 2d. A well-ordered honeycomb-like concave patterned morphology can be seen in most of the area; however, slight deviation from ordered morphology is also present in some small area. The pores are arranged in perfect hexagonal ordered in a very large domain area. The concave shape morphology is perfectly matched with the convex shape morphology of bottom surface of TNT (Figure 2b).

### The second-step anodization in EG-based electrolyte

**Figure 3:**
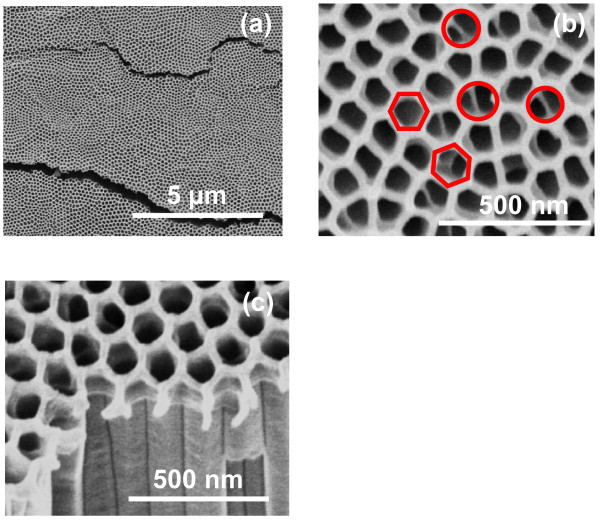
**FESEM images of TiO_2 _nanotubes fabricated in EG containing 0.5 wt% NH_4_F and 0.2 wt% H_2_O via second-step anodization**: **(a) **top surface view at low magnification, **(b) **top surface view at high magnification, **(c) **cross-sectional view.

The top and the cross-sectional surface morphologies of TNT obtained after the second-step anodization in EG-based electrolyte are shown in Figure 3. The top surface topologies of the TiO_2 _nanotubes at a low- and a high-magnification are shown in Figure 3a,b, respectively, without a post-anodizing treatment. Highly ordered TiO_2 _nanotube arrays with open mouths are clearly visible in the images in spite of 20 h anodization. This is attributed to the honeycomb-like patterned morphology of Ti-substrate (Figure 2d), which not only protects the TiO_2 _nanotubes from sealing and bundling but also produces TiO_2 _nanotubes with uniform heights. The honeycomb-like patterned morphology of individual hexagonal ring is clearly reflected in the magnified image (hexagonal marked pores in Figure 3b); however, the hexagonal shape geometry of individual concave nano-dimples is slightly distorted in some area due to a longer anodization time. The formation of a thin nano-porous layer on the top surface of TiO_2 _nanotubes is evident from the areas marked with circles, where nanotubes wall can be clearly seen inside nanopores. This result is also verified from the cross-sectional image of the nanotubes (Figure 3c), where nanotubes are connected with each other via a thin nanoporous layer. These results indicate that the formation of nanotubes is initiated exactly below the honeycomb-like patterned morphology during the second-step anodization and act as a template for further growth of nanotubes; however, appearance of the nanotubes wall inside the nanopores (Figure 3b) also suggests slight deviations. These results also reveal that nanopores have almost uniform diameters and that nanotubes walls are very smooth throughout their entire lengths.

### The second-step anodization in HF-based aqueous electrolyte

**Figure 4:**
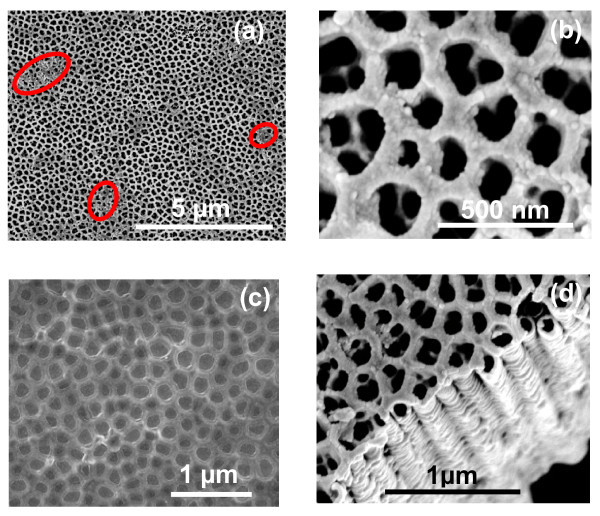
**FESEM images of TiO_2 _nanotubes fabricated in 0.5 wt% aqueous-based HF electrolyte via second-step anodization**: **(a) **top surface view at low magnification, **(b) **top surface view at high magnification, **(c) **top surface view of patterned Ti-substrate anodized for 10 min, and **(d) **cross-sectional view.

The surface and the cross-sectional topologies of TiO_2 _nanotubes obtained after the second-step anodization in HF-containing aqueous electrolyte is shown in Figure 4. The top surface view of the TiO_2 _nanotubes at a low- and a high-magnification is shown in Figure 4a,b, respectively. Irregular shape of pores can be seen clearly in the images. These images show that the honeycombs-like pre-patterned morphology and the hexagonal shape geometry of the individual nanodimples (Figure 2d) are completely destroyed after the second-step anodization unlike EG-based electrolyte. This is due to the strong dissolution power of the HF-based electrolyte where TiO_2 _dissolution is very fast compared to the EG electrolyte [28]. The dissolution power of the HF-based electrolyte is evident from Figure 4c, which shows the top surface morphology of the pre-patterned Ti-substrate after 5-10 min of anodization. Even after a very short anodizing time, the original pre-patterned hexagonal shape morphology of Ti-substrate (Figure 2d) is completely vanished and a new shape morphology emerged. The new morphology is retained in most of the area, however, in some places (marked area in Figure 4a), the nanopores are dissolved and led to the covering of TNT at the top surface. This is attributed to the extended anodization in HF-based electrolytes. The surface image (Figure 4b) and the cross-sectional image (Figure 4d) reveal the formation of a thin nanoporous layer on the top surface of TNT and show the roughness of TNT walls. Some of the nanopores covering two and more nanotubes can also be seen, which confirms the formation of a nanoporous layer on the top surface of TNT. The roughness of the nanotube walls is ascribed to water in the electrolyte [21].

### The second-step anodization in glycerol-based electrolyte and formation of TiO_2 _nano-porous structures

**Figure 5:**
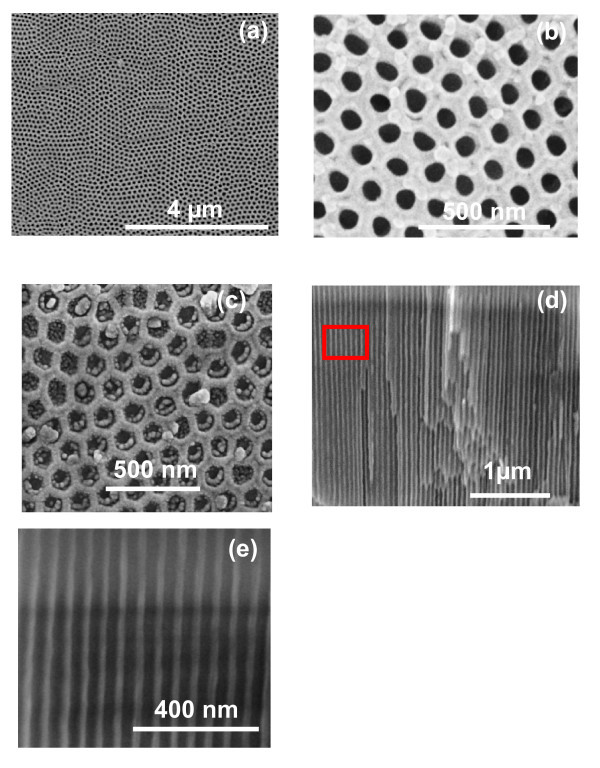
**FESEM images of TiO_2 _nanotubes fabricated in glycerol containing 0.5 wt% NH_4_F and 0.2 wt% H_2_O via second-step anodization**: **(a) **top surface view at low magnification, **(b) **top surface view at high magnification, **(c) **top surface view of patterned Ti-substrate anodized for 10 min, **(d) **cross-sectional view at low magnification, **(e) **cross-sectional view of the marked area at high magnification.

**Figure 6:**
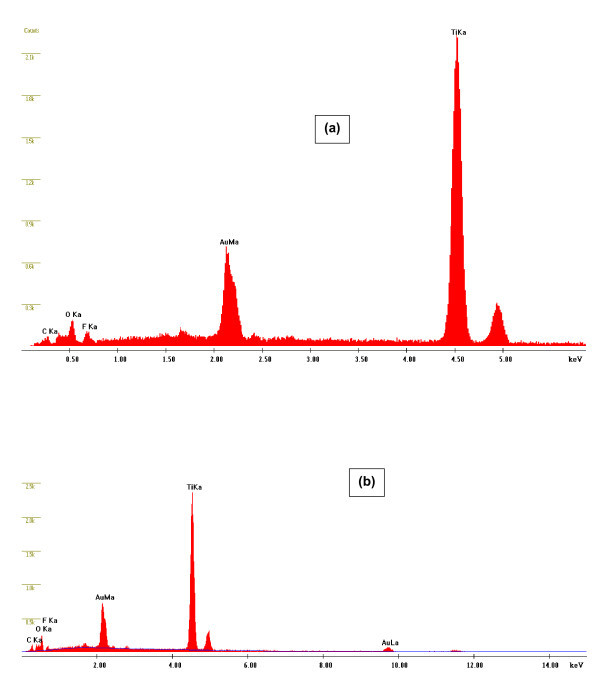
**Energy dispersive X-ray spectroscopy (EDX) spectra of TNP, (a) top and, (b) bottom surfaces**.

A complete TNP structure was obtained when a pre-patterned Ti-substrate, obtained in EG-based electrolyte via first-step anodization, is secondly anodized in glycerol-based electrolyte. Figure 5a,b shows the top surface view of the TNP structure at a low- and a high-magnification, respectively, without post-anodizing treatment. It is evident from these images that the nanopores are very clear, regular, uniform, and highly-oriented. The average pore diameter is approximately 70 nm, while the inter-pore distance (distance between centers of the pores) is about 130 nm. It is important to note that the hexagonal shape of original pre-patterns dimples of honeycomb-like morphology (Figure 2d) is converted in to a circular-like shape during the second-step anodization in glycerol. This kind of morphology has been reported for pre-patterned Al and Ti during anodization [28] and ascribed to a long anodization time. However, we assume that this kind of circular shape morphology is also due to the viscosity of the electrolyte. It has been reported that pore diameters of nanotubes also depend upon the nature of electrolyte [23] as well as the anodization potential and the anodizing time [21]. An electrolyte with a high viscosity will produce nanopores/nanotubes with small diameters and vice versa. This is clearly evident from Figure 5c, which shows the top surface morphology of the pre-patterned Ti-substrate after 5-10 min of the second-step anodization in glycerol-based electrolyte. Since the viscosity of glycerol is 945 cP at 25°C while that of EG is 16 cP at 25°C [33], therefore, the pore diameter will be smaller in glycerol as compared to that in EG. It is because of this fact that growth of nanopores start within the honeycomb-like hexagonal pre-patterned ring during the second-step anodization in glycerol-based electrolyte and resulted in a smaller pore diameter with circular shape morphology. The thickness of honeycomb-like patterned hexagonal rings is also greater after the second-step anodization in glycerol compared to the thickness of original honeycomb-like hexagonal pre-pattern before the second-step anodization (Figure 2d). This result further supports our assumption about the growth of nanopores within the original honeycomb-like hexagonal pattern ring morphology during the second-step anodization in glycerol-based electrolyte. The cross-sectional morphology at a low- and a high-magnification is shown in Figure 5d,e, respectively. Uniform and parallel nano-channels can be clearly seen in these micrographs. The width of the nano-channel is approximately 70 nm, while the inter channel distance is approximately 130 nm which is matched well with the top surface morphology of the TNP. This kind of parallel channel morphology has been reported in the literature for TNP structure [34]. Very recently Schmuki and co-workers [31] also reported a TNP structure. According to their findings, the formation of TNP structure is due to the optimized content of water in the electrolyte which suppresses the dissolution of F-rich layer in the cell boundaries. F-rich layer is always present at the bottom of TiO_2 _nanotubes as well as at the cell boundaries. Energy dispersive X-ray spectroscopy (EDX) analysis (Figure 6; Table 1) of the top and the bottom surface of TNP structure is in line with the literature [35]. Significant amount of C and F is also found besides Ti and O. The presence of F-rich layer in the boundaries between the cells is essential for the formation of nano-porous structure. According to Stokes-Einstein relation, the diffusion coefficient is inversely proportional to the viscosity of the electrolyte. Since the viscosity of glycerol is approximately 60 times higher than EG at 25°C, the diffusion of H^+ ^is expected to be reduced in glycerol during anodization and thus H^+ ^cannot diffuse easily in the cell boundaries. This will protect F-rich layer between the cell boundaries from dissolution and hence resulted in the formation of nano-porous structure. As a consequence, F-rich layer in the cell boundaries can be protected from dissolution which led to the formation of nano-porous structure. This is evident from the content of F in the top and bottom surface of TNP in the EDX analysis (Table 1). However, dissolution of the F-rich layer between the cells boundaries results in the formation of the nanotubular structure [31].

**Table 1:** Energy dispersive X-ray spectroscopy (EDX) analysis of top and bottom surface of TNP

Element	Top		Bottom	
	wt%	at%	wt%	at%
C K	1.9	5.16	2.86	7.35
O K	14.92	30.49	19.04	36.71
F K	7.31	12.58	5.78	9.39
TiK	75.87	51.77	72.31	46.56
Total	100	100	100	100

### Effect of anodizing time on the morphologies of TNT and TNP

**Figure 7:**
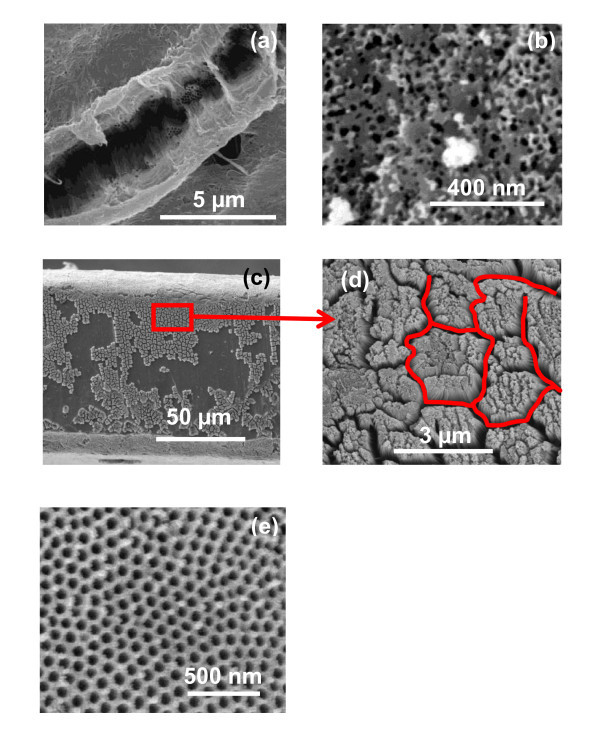
**FESEM images of TNT fabricated in EG containing 0.5 wt% NH_4_F and 0.2 wt% H_2_O via single-step anodization for different times**: **(a) **top surface view after 72 h of anodization, **(b) **top surface view after 11 h of anodization, **(c) **top surface view after ultrasonic agitation of 20 min in DI H_2_O, **(d) **magnified image of the marked area of (c), and **(e) **top surface view of TNP structure obtained after prolonged anodizing time (72 h) via second-step anodization in glycerol-based electrolyte.

In order to study the effect of anodizing time on surface topologies of TNT and TNP structure after the first- and the second-step anodization, a set of experiments were carried at different anodizing times. We found that generally the top surface of TiO_2 _nanotubes is always covered with some kind of oxide flakes irrespective of the anodizing time. Figure 7a shows the top surface morphology of TNT obtained via the first-step anodization of 72 h in EG-based electrolyte. Formation of nanorods on the top surface of TNT is clearly evident from the image. It is well-known fact that extended anodization time led to the wall thinning of already formed nanotubes at the top surface due to the chemical dissolution. The nanotubes are collapsed and disintegrated at the surface, thus, covering the top of nanotubes. This kind of morphology has been reported in the literature for TNTs [36]. The nanotubes are also buried under the oxide flakes, when the anodization is carried out in the same electrolyte even for a short time (11 h) as shown in the Figure 7b. The oxide clumps (nanorods and flakes) on the surface can be removed with the help of ultrasonication with optimized time duration. It is worth mentioning that severe ultrasonic agitation led to the partial removal of TiO_2 _nanotubes from the underlying Ti-substrate, as shown in Figure 7c. The partial removal of TiO_2 _nanotubes might be attributed to the compressive stresses generated in the barrier layer between the nanotubes and the Ti-foil during ultrasonic agitation. The barrier layer has lower mechanical strength as compared to Ti; so compressive stresses in the barrier layer will lead to the partial removal of TiO_2 _nanotubes from the underlying Ti-substrate. Figure 7d represents the high-magnification image of the marked area in Figure 7c. It is clear that ultrasonic agitation may also produce bundling issues (marked area of Figure 7d). These results suggest that the second anodization is necessary to obtain open tube morphology with a uniform height throughout the entire sample without the bundling problem, which can be used as a template for easy deposition of secondary materials [37]. In order to see the effect of prolonged anodizing time on the pore morphology after the second-step anodization, another experiment on pre-patterned Ti-substrate was performed in glycerol-based electrolyte for 72 h. The top surface morphology of TNP structure obtained after 72 h anodization is shown in Figure 7e without further processing. It is clear from the image that TNP structure is retained even after a prolonged anodizing time and the nanopores are arranged more regularly when compared to a short anodizing time. Thus, prolonged anodizing time improves the pore ordering to a great extent [5]. However, the surface is not very much clean and some debris can be clearly seen in the image. The debris can be removed easily with the help of an optimized ultrasonic agitation.

## Conclusions

In summary, we have fabricated a complete titania nanoporous structure with uniform and parallel nanochannels using a two-step anodization process. The average pore diameter was approximately 70 nm and inter-pore distance was approximately 130 nm. Self-organized, highly ordered, and very smooth TNTs were fabricated in EG-based electrolyte by the first-step anodization. The top surfaces of TNTs were covered with an oxide layer irrespective of the anodizing time. Clean and homogeneous honeycomb-like patterned Ti substrates were left off after the detachment of TNTs from the underlying Ti-foil. The second-step anodization on the patterned Ti-substrate produced a uniform and closed packed TNTs with open end morphology. The second-step anodization in EG and aqueous HF-based electrolytes produced TNTs covered with a thin nanoporous layer on the top. Very rough and disordered morphology of TNTs were obtained in HF-based electrolyte unlike EG-based electrolyte via the second-step anodization. A highly oriented and complete TNP structure was obtained when the second-step anodization was conducted in glycerol-based electrolyte. TNP structure were attributed to the suppression of F-rich layer dissolution between the cell boundaries in the viscous electrolyte. In addition, we found that TNP structure retained in shape even in spite of a long anodizing time (72 h) after the second-step anodization and that its ordering was improved to a great extent. This study provides a simple route to fabricate highly oriented TNPs with parallel and uniform nanochannels, which may be useful for high performance applications such as sensors, filters, dye sensitized solar cells, and photocatalysis.

## Abbreviations

ATO: anodic titanium oxide; DI: deionized; EDX: energy dispersive X-ray spectroscopy; EG: ethylene glycol; FESEM: field emission scanning electron microscope; PEG: polyethylene glycol; PAA: porous anodic alumina; TNP: titania nano-porous.

## Competing interests

The authors declare that they have no competing interests.

## Authors' contributions

GA presided over and fully participated in all of the work. CC and JK participated in the preparation of the samples. SY helped in characterization of the samples. SC give the idea of the study and finalize the manuscript. All authors read and approved the final manuscript.
